# ﻿*Tenkana*, a new genus of jumping spiders (Salticidae, Plexippina) from South Asia, with the new Indian species *Tenkanajayamangali*

**DOI:** 10.3897/zookeys.1215.133522

**Published:** 2024-10-11

**Authors:** Kiran Marathe, John T. D. Caleb, Wayne P. Maddison, B. G. Nisha, Chinmay C. Maliye, Y. T. Lohit, Krushnamegh Kunte

**Affiliations:** 1 Department of Zoology and Beaty Biodiversity Museum, University of British Columbia, 6270 University Boulevard, Vancouver, British Columbia, V6T 1Z4, Canada; 2 National Centre for Biological Sciences, Tata Institute of Fundamental Research, GKVK Campus, Bengaluru, 560065, India; 3 Department of Anatomy, Saveetha Medical College & Hospitals, Saveetha Institute of Medical and Technical Sciences, Saveetha University, Chennai 602105, Tamil Nadu, India; 4 Departments of Zoology and Botany and Beaty Biodiversity Museum, University of British Columbia, 6270 University Boulevard, Vancouver, British Columbia, V6T 1Z4, Canada; 5 Chilipili, 4th Main 5b Right cross Jayanagar West, Tumakuru, 572102, Karnataka, India; 6 Wildlife Aware Nature Club, Tumakuru 572101, Karnataka, India; 7 146, Sunkalpalya, Bengaluru 560060 Karnataka, India; 8 Renusri Nilaya, 2nd ‘A’ cross, CSI layout, Tumakuru 572102, Karnataka, India

**Keywords:** Araneae, biodiversity research, classification, new combination, phylogenomics, systematics, taxonomy, ultraconserved elements

## Abstract

We describe a new plexippine genus, *Tenkana***gen. nov.**, supported by phylogenomic data from ultraconserved elements (UCEs), Sanger sequences of four genes, and morphological evidence. The type species, *Tenkanamanu* (Caleb, Christudhas, Laltanpuii & Chitra, 2014), **comb. nov.** is transferred from *Colopsus*, as is *Tenkanaarkavathi* (Caleb, 2022), **comb. nov.** The phylogenomic data places *Tenkana* among the plexippines near *Hyllus* C.L. Koch, 1846 and *Telamonia* Thorell, 1887, while the constrained four-gene phylogeny indicates that *Tenkana* is distinct from *Colopsus*. Additionally, we describe a new species, *Tenkanajayamangali***sp. nov.**

## ﻿Introduction

The current composition of the jumping spider genus *Colopsus* Simon, 1902 is questionable due to morphological inconsistency among species. The genus now holds two disparate groups: (i) the *cancellatus* species group, consisting of vegetation-dwelling forest species mostly known from Sri Lanka, recognizable by the male palp lacking a tegular lobe and the body glossy and elongate; and (ii) the *manu* species group, consisting of ground-dwelling species found in Sri Lanka and relatively drier regions of southern India, recognizable by a conspicuous tegular lobe and a more compact body, neither glossy nor elongate. The type species, *Colopsuscancellatus* Simon, 1902, is in the first group. When Kanesharatnam and Benjamin (2021) revived the genus *Colopsus*, previously synonymized with *Evarcha* Simon, 1902 (Prószyński 1984), they described three glossy species in the *cancellatus* group. However, they also placed in *Colopsus* a non-glossy compact-bodied species, *C.cinereus*, based on a male. They had molecular evidence to place the glossy species phylogenetically, but not *C.cinereus*, whose inclusion in *Colopsus* was considered provisional due to the lack of female morphology and molecular evidence. Subsequently, Caleb et al. (2022) transferred *Hyllusmanu* (Caleb, Christudhas, Laltanpuii & Chitra, 2014) to *Colopsus* and synonymized *C.cinereus* with *C.manu*. Thus, currently, *Colopsusarkavathi* Caleb, 2022 and *C.manu* (Caleb, Christudhas, Laltanpuii & Chitra, 2014) represent the *manu* group, and *C.cancellatus*, *C.ferruginus* Kanesharatnam & Benjamin, 2021, *C.magnus* Kanesharatnam & Benjamin, 2021, and *C.tenuesi* Kanesharatnam & Benjamin, 2021 represent the *cancellatus* group.

Our goal here is to resolve the phylogenetic placement of the two groups. We used ultraconserved element (UCE) data from the *manu* group to test its monophyly and to explore its relationships among plexippines. UCE data is unavailable for the type species of *Colopsus* and other Sri Lankan species, but Kanesharatnam and Benjamin (2021) obtained data for four genes from them. We combined their data with data for the same genes obtained as bycatch from UCE work, in order to determine the placement of the *cancellatus* species group relative to the *manu* species group.

The molecular, morphological, and ecological evidence we present justify establishing a new genus comprising ground-dwelling species of southern India and Sri Lanka. We describe, diagnose, and illustrate a new species within this genus.

## ﻿Materials and methods

### ﻿Materials examined

The Indian specimens examined in this study are deposited in two repositories. (i) The male holotype (I/SP-48) and a female paratype (I/SP-49) in the Southern Regional Centre, Zoological Survey of India (**ZSIC**), Chennai, Tamil Nadu, India and (ii) three paratypes (1 ♂ and 2 ♀♀) in the Biodiversity Lab Research Collections of the National Centre for Biological Sciences (**NCBS**), Bengaluru, India (http://biodiversitycollections.in/). Individual specimens deposited in NCBS are identified by three-digit voucher codes prefixed with “IBC-BX”. Non-Indian specimens are deposited in the University of British Columbia Spencer Entomological Collection.

### ﻿Morphology

We examined and photographed 70% ethanol-preserved specimens using a Leica MC190 HD camera attached to a Leica SAPO stereomicroscope automatically using the Leica Application Suite (LAS) v. 4.13. We examined and photographed the genitalia using a Leica MC190 HD camera attached to a Leica DM3000 LED compound microscope and prepared the drawings by digitally tracing the photographs. We photographed the living spiders with a Tamron 90 mm macro lens attached to a Nikon Z6 camera and a Godox flash covered with a Radiant Diffuser.

Descriptions of colour patterns are based on ethanol-preserved specimens. The male genitalic description is based on the left palp. Carapace length is measured from the base of the anterior median eyes to the posterior margin of the carapace medially, while abdomen length is measured from the anterior to the end of the anal tubercle. All measurements are in millimetres. Leg measurements are represented as follows: total length (femur, patella, tibia, metatarsus, and tarsus). Abbreviations used here are as follows: **ALE**, anterior lateral eye; **ECP**, epigynal coupling pocket; **PME**, posterior median eye; **RTA**, retrolateral tibial apophysis.

### ﻿Taxon sampling for phylogenomic analysis

The morphology (conspicuous tegular lobe and two ECPs) of the species of *Tenkana* is consistent with their current placement among the plexippines. Therefore, we gathered molecular data for *T.arkavathi*, *T.jayamangali*, and *T.manu* and appended it to (Marathe et al. 2024b) plexippine-biased UCE phylogenomic dataset, which included 16 plexippines, five outgroups (three harmochirines, one salticine, and one chrysilline). We also included cf. *Colopsus* sp. from Lin et al. (2024) to allow cf. *Colopsus*, along with *Pancorius* from the Marathe et al. (2024b) dataset, a chance to capture *Tenkana* in the phylogenomic analysis. The total set of 25 species comprising 20 plexippines and five outgroups used in the phylogenomic analysis, with their taxonomic authorities indicated, is listed in Table 1.

**Table 1.** Specimens used in UCE phylogenomic and four-gene phylogenetic analysis. The sex, latitude, and longitude information for ‘IFS_SAL_#’ vouchers are inferred from Kanesharatnam and Benjamin (2021).

Species	Voucher	Sex	Locality	Lat, long
*Anarrhotusfossulatus* Simon, 1902	AS19.1319	♂	Singapore	1.379, 103.816
*Artabruserythrocephalus* (C.L. Koch, 1846)	AS19.2205	♂	Singapore	1.355–7, 103.774–5
*Baryphasahenus* Simon, 1902	d536	♂	South Africa	-25.95, 30.56
*Bianormaculatus* (Keyserling, 1883)	NZ19.9864	♂	New Zealand	-42.1691, 172.8090
*Carrhotus* sp.	AS19.4650	♂	India	12.2145, 75.653–4
cf. *Colopsus* sp.	JXZ795	♀	China	21.910897, 101.283422
*Colopsuscancellatus*	IFS_SAL_360	♀	Sri Lanka	6.843333, 80.677778
*Colopsuscancellatus*	IFS_SAL_797	?	Sri Lanka	7.746111, 80.131667
*Colopsusferruginus*	IFS_SAL_233	?	Sri Lanka	7.859444, 80.674444
*Colopsusferruginus*	IFS_SAL_248	♂	Sri Lanka	7.298333, 80.641389
*Colopsusmagnus*	IFS_SAL_832	♂	Sri Lanka	7.145833, 80.698056
*Colopsusmagnus*	IFS_SAL_906	?	Sri Lanka	6.766667, 80.6
*Chysillavolupe* (Karsch, 1879)	AS19.6089	♂	India	12.223, 76.627
*Epeus* sp.	DDKM21.055	♂	Singapore	1.355, 103.78
*Evacinbulbosa* (Żabka, 1985)	AS19.2123	♂	Singapore	1.406, 103.971
*Evarchafalcata* (Clerck, 1757)	RU18-5264	♂	Russia	53.721, 77.726
*Ghatippuspaschima* Marathe & Maddison, 2024	IBC-BP833	♂	India	12.220–1, 75.657–8
*Habronattushirsutus* (Peckham & Peckham, 1888)	IDWM.21018	♂	Canada	48.827, -123.265
*Hylluskeratodes* (van Hasselt, 1882)	DDKM21.028	♂	Malaysia	3.325, 101.753
*Hyllussemicupreus* (Simon, 1885)	AS19.4415	♂	India	12.2156, 75.6606
*Iranattusrectangularis* Prószyński, 1992	DDKM21.091	juv.	India	26.28, 70.40
*Pancoriusdentichelis* (Simon, 1899)	SWK12-0042	♂	Malaysia	1.605–6, 110.185–7
*Pancoriusalboclypeus* Kanesharatnam & Benjamin, 2021	IFS_SAL_1145	♂	Sri Lanka	7.338611, 80.850833
*Pancoriusaltus* Kanesharatnam & Benjamin, 2021	IFS_SAL_1074	?	Sri Lanka	—
*Pancoriusathukoralai* Kanesharatnam & Benjamin, 2021	IFS_SAL_1048	♂	Sri Lanka	7.145833, 80.698056
*Pancoriuspetoti* Prószyński & Deeleman-Reinhold, 2013	SWK12-0195	♂	Malaysia	1.603–4, 110.185
*Pelleneslimbatus* Kulczyński, 1895	RU18-5679	♂	Russia	50.0501, 89.3878
*Plexippuspaykulli* (Audouin, 1826)	AS19.7337	♂	India	12.825–6, 78.252–3
*Ptocasiusweyersi* Simon, 1885	DDKM21.069	♂	Singapore	1.36, 103.78
*Telamoniafestiva* Thorell, 1887	DDKM21.048	♂	China	21.8105, 107.2925
*Tenkanaarkavathi* (Caleb, 2022)	IBC-BX509	♂	India	13.327, 77.657
*Tenkanajayamangali* **gen et. sp. nov.**	IBC-BX511	♀	India	13.3843, 77.2069
*Tenkanamanu* (Caleb, Christudhas, Laltanpuii & Chitra, 2014)	IBC-BX510	♀	India	12.0305, 79.8483
*Thyeneimperialis* (Rossi, 1846)	AS19.6443	♂	India	12.216, 76.625

### ﻿Taxon sampling for the four-gene phylogenetic analysis

To test *Tenkana*’s placement relative to the type species of *Colopsus* and others from Sri Lanka, we constructed matrices of mitochondrial cytochrome oxidase I (COI) and nuclear 28S, 18S and Histone 3 (H3) from publicly available data for *Colopsus* from Kanesharatnam and Benjamin’s (2021) study. We appended bycatch data for the same four gene regions present among the sequence capture genomic assemblies from the UCE dataset to the four gene matrices of *C.cancellatus*, *C.ferruginus*, and *C.magnus*. We followed a bycatch protocol similar to that described by Maddison et al. (2020), constructing local BLAST databases from SPAdes (Nurk et al. 2013) assemblies of each taxon in the UCE dataset. These assemblies were queried with publicly available COI, 28S, 18S, and H3 sequences from seven different salticid species (Aelurilluscf.ater (Kroneberg, 1875), *Bianormaculatus* (Keyserling, 1883), *Colopsuscancellatus*, *Colopsusferruginus*, *Hyllustreleaveni* Peckham & Peckham, 1902, *Pancoriusathukoralai* Kanesharatnam & Benjamin, 2021, and *Salticiusscenicus* (Clerck, 1757)). Thus, the total set of 31 taxa (25 UCE bycatch and six Sri Lankan *Colopsus* of three species) are used in the four-gene phylogenetic analysis. The accession numbers are listed in Table 3.

### ﻿Ultraconserved element (UCE) data

Molecular data was gathered for UCE loci using target enrichment sequencing methods (Faircloth 2017), using the RTA_v3 probeset (Zhang et al. 2023) and following the protocols of Marathe et al. (2024a).

Raw demultiplexed reads were processed with PHYLUCE v. 1.6 (Faircloth 2016), quality control and adapter removal were performed with Illumiprocessor wrapper (Faircloth 2013), and assemblies were created with SPAdes v. 3.14.1 (Nurk et al. 2013) using options at default settings. The UCE loci were recovered using RTA_v3 probeset (Zhang et al. 2023). The recovered loci were aligned with MAFFT using L-INS-i option (Katoh and Standley 2013). The aligned UCE loci were then trimmed with Gblocks (Castresana 2000; Talavera and Castresana 2007) using –b1 0.5, –b2 0.7, –b3 8, –b4 8, –b5 0.4 setting within Mesquite v. 3.81 (Maddison and Maddison 2023b). As in the analysis of Maddison et al. (2020), loci suspected to include paralogies were deleted based on branch lengths in RAxML (Stamatakis 2014) inferred gene trees. Loci represented in fewer than 10 taxa total were deleted.

### ﻿UCE phylogenomic analysis

Maximum-likelihood phylogenetic and bootstrap analyses were performed with IQ-TREE v. 2.3.4 (Minh et al. 2020) using the Zephyr v. 3.31 package (Maddison and Maddison 2023a) in Mesquite v. 3.81 (Maddison and Maddison 2023b) on the concatenated, unpartitioned UCE dataset with 25 taxa. For the phylogenetic tree inference, the option -m TEST (standard model selection followed by tree inference, edge-linked partition model, no partition-specific rates) was used with 10 search replicates. For the bootstrap analysis, a single IQ-TREE search was used for each of the 1000 search replicates.

### ﻿Four-gene phylogenetic analysis

The loci were aligned using MAFFT with the L-INS-i option, partitioned by locus, assigned codon positions to minimize stop codons for H3 and COI, and then concatenated in Mesquite v. 3.81. Maximum-likelihood phylogenetic and standard bootstrap analyses were performed with IQ-TREE v. 2.3.4 on the concatenated dataset using the Zephyr v. 3.31 package in Mesquite v. 3.81. The option -m MFP+MERGE (find the best partition scheme including FreeRate heterogeneity followed by tree inference, edge-linked partition model (-spp), with partition-specific rates) was used with 10 search replicates. For the bootstrap analysis, a single IQ-TREE search was used for each of 1000 search replicates. For both, four partitions were provided. IQ-TREE found the best partition scheme (Chernomor et al. 2016) and best fitting models (Kalyaanamoorthy et al. 2017) for phylogenetic tree inference: GTR+F+I+R2 model for 28S+H3 merged partition, GTR+F+I+G4 for 18S and COI separate partitions. Two trees were obtained: (1) unconstrained tree for 34 taxa, followed by standard bootstrap analysis (Fig. 3). (2) A constrained tree for the same dataset, followed by standard bootstrap analysis with an additional option of -g to specify (*Tenkana*, (*Hyllus*, *Telamonia*)) as the topological constraint (Fig. 2).

### ﻿Data availability

The raw sequence reads obtained from UCE capture are stored within the Sequence Read Archive (BioProject: PRJNA1145028, https://www.ncbi.nlm.nih.gov/bioproject/PRJNA1145028), and their accession numbers are listed in Table 2. The sequences obtained through UCE bycatch are available from the nucleotide database of the National Center for Biotechnology Information (NCBI, https://www.ncbi.nlm.nih.gov/), and their accession numbers are listed in Table 3. Concatenated UCE and legacy Sanger four-gene matrices used for phylogenetic and bootstrap analysis, along with trees, are available on the Dryad data repository (https://doi.org/10.5061/dryad.b8gtht7np).

**Table 2.** Specifics of molecular data used for this phylogenomic analysis. Molecular data was generated based on RTA_v2 probeset. “**SRA**” is Sequence Read Archive accession number available through NCBI; “**Reads pass QC**” is the number of reads after the removal of adapter-contamination and low-quality bases using Illumiprocessor; “**Total UCE loci**” is the total number of UCE loci recovered with RTA_v2 probeset; “**After paralogy filter**” is the number of UCE loci after deletion of suspected paralogous loci based on branch length ratios; “**In at least 10 taxa**” is the number of UCE loci in at least 10 or more taxa after branch length criteria; “**Filtered UCE sequence length**” is the concatenated sequence length of filtered UCE loci; “**Total loci**” is the number of UCE loci represented among all taxa. An asterisk besides cf. *Colopsus* sp. indicates that the SPAdes assembly was obtained from the senior author of Lin et al. (2024).

Species	Voucher	SRA	Reads pass QC	Total UCE loci	After paralogy filter	In at least 10 taxa	Filtered UCE sequence length
*Anarrhotusfossulatus*	AS19.1319	SRR27728361	15542927	2525	2442	2359	1937986
*Artabruserythrocephalus*	AS19.2205	SRR27728359	14903498	2839	2753	2705	2160708
*Baryphasahenus*	d536	SRR27728358	2653688	2256	2183	2171	940794
*Bianormaculatus*	NZ19.9864	SRR27728369	7914005	2963	2871	2764	2228134
*Carrhotus* sp.	AS19.4650	SRR27728370	5272657	2922	2834	2751	2179446
*cf. *Colopsus* sp.	JXZ795	SRR27541609	NA	2566	2478	2421	1977579
*Chrysillavolupe*	AS19.6089	SRR28802507	4968344	2878	2787	2692	2154458
*Epeus* sp.	DDKM21.055	SRR27728357	13896435	2898	2809	2743	2252399
*Evacinbulbosa*	AS19.2123	SRR27728356	10851810	2767	2683	2598	2014108
*Evarchafalcata*	RU18-5264	SRR27728355	11538276	2763	2676	2629	2064600
*Ghatippuspaschima*	IBC-BP833	SRR27728354	7881860	2893	2806	2748	2249548
*Habronattushirsutus*	IDWM.21018	SRR27728360	6581974	2821	2734	2647	2046288
*Hylluskeratodes*	DDKM21.028	SRR27728353	11349372	2926	2831	2749	2233053
*Hyllussemicupreus*	AS19.4415	SRR27728368	9874003	2942	2852	2784	2249371
*Iranattusrectangularis*	DDKM21.091	SRR28802508	14825117	2927	2839	2767	1863497
*Pancoriusdentichelis*	SWK12-0042	SRR27728367	6025337	3092	3003	2931	2167369
*Pancoriuspetoti*	SWK12-0195	SRR27728366	5116119	2980	2891	2820	2147865
*Pelleneslimbatus*	RU18-5679	SRR28802506	4288156	2661	2577	2522	1876069
*Plexippuspaykulli*	AS19.7337	SRR27728365	7445183	2931	2839	2764	2048859
*Ptocasiusweyersi*	DDKM21.069	SRR27728364	9926900	2880	2796	2739	2166828
*Telamoniafestiva*	DDKM21.048	SRR27728363	7908436	2950	2856	2797	2281787
*Tenkanaarkavathi*	IBC-BX509	SRR30215970	3427028	2723	2639	2618	2126265
*Tenkanajayamangali*	IBC-BX511	SRR30215969	2496709	2829	2740	2714	2230595
*Tenkanamanu*	IBC-BX510	SRR30215968	3553397	2692	2600	2578	1979121
*Thyeneimperialis*	AS19.6443	SRR27728362	7797854	2893	2802	2733	2232707
			Average:	2820.68	2732.84	2669.76	2072377.36
			Minimum:	2256	2183	2171	940794
			Maximum:	3092	3003	2931	2281787
			**Total loci**:	3404	3302	3043	2557548

**Table 3.** Accession numbers of nuclear 28S, 18S, H3, and mitochondrial COI fragments used in the four-gene phylogenetic analysis. An asterisk beside species indicates that the data for those were downloaded from NCBI and NA in cells indicate that data is not available.

Species	Voucher	28S	18S	H3	COI
*Anarrhotusfossulatus*	AS19.1319	PQ278946	PQ278921	PQ273890	PQ305882
*Artabruserythrocephalus*	AS19.2205	PQ278958	PQ278933	PQ273902	PQ305894
*Baryphasahenus*	d536	PQ278949	PQ278924	PQ273893	PQ305885
*Bianormaculatus*	NZ19.9864	PQ278944	PQ278931	PQ273888	PQ305892
*Carrhotus* sp.	AS19.4650	PQ278960	PQ278935	PQ273904	PQ305896
cf. *Colopsus* sp.	JXZ795	PQ278963	PQ278938	PQ273907	NA
*Chrysillavolupe*	AS19.6089	PQ278959	PQ278934	PQ273903	PQ305895
*Colopsuscancellatus	IFS_SAL_360	MN888680.1	MN888692.1	MN895432.1	MN895417.1
*Colopsuscancellatus	IFS_SAL_797	MN888677.1	MN888691.1	NA	MN895414.1
*Colopsusferruginus	IFS_SAL_233	MN888672.1	MN888689.1	MN895429.1	MN895411.1
*Colopsusferruginus	IFS_SAL_248	MN888673.1	MN888690.1	MN895431.1	MN895409.1
*Colopsusmagnus	IFS_SAL_832	MN888671.1	MN888687.1	NA	MN895408.1
*Colopsusmagnus	IFS_SAL_906	MN888670.1	MN888686.1	NA	MN895407.1
*Epeus* sp.	DDKM21.055	PQ278945	PQ278920	PQ273889	PQ305881
*Evacinbulbosa*	AS19.2123	NA	PQ278927	PQ273896	PQ305888
*Evarchafalcata*	RU18-5264	PQ278955	PQ278930	PQ273899	PQ305891
*Ghatippuspaschima*	IBC-BP833	PQ278962	PQ278937	PQ273906	PQ305897
*Habronattushirsutus*	IDWM.21018	PQ278948	PQ278923	PQ273892	PQ305884
*Hylluskeratodes*	DDKM21.028	PQ278942	PQ278917	PQ273887	PQ305879
*Hyllussemicupreus*	AS19.4415	PQ278947	PQ278922	PQ273891	PQ305883
*Iranattusrectangularis*	DDKM21.091	PQ278953	PQ278928	PQ273897	PQ305889
*Pancoriusalboclypeus	IFS_SAL_1145	MN888667	MN888685	MN895424	MN895404
*Pancoriusaltus	IFS_SAL_1074	MN888666	NA	MN895422	MN895403
*Pancoriusathukoralai	IFS_SAL_1048	MN888663	MN888683	MN895421	MN895401
*Pancoriusdentichelis*	SWK12-0042	PQ278957	PQ278932	PQ273901	PQ305893
*Pancoriuspetoti*	SWK12-0195	PQ278939	PQ278914	PQ273884	PQ305876
*Pelleneslimbatus*	RU18-5679	PQ278940	PQ278915	PQ273885	PQ305877
*Plexippuspaykulli*	AS19.7337	PQ278941	PQ278916	PQ273886	PQ305878
*Ptocasiusweyersi*	DDKM21.069	PQ278961	PQ278936	PQ273905	NA
*Telamoniafestiva*	DDKM21.048	PQ278954	PQ278929	PQ273898	PQ305890
*Tenkanaarkavathi*	IBC-BX509	PQ278950	PQ278925	PQ273894	PQ305886
*Tenkanajayamangali*	IBC-BX511	PQ278943	PQ278918	NA	NA
*Tenkanamanu*	IBC-BX510	PQ278956	PQ278919	PQ273900	PQ305880
*Thyeneimperialis*	AS19.6443	NA	PQ278926	PQ273895	PQ305887

## ﻿Results

### ﻿Phylogenetic results

Table 2 lists the sequence data recovered from the 25 taxa. 3404 UCE loci were initially recovered. Of these, 3302 remained after removing those suspected to include paralogies on branch lengths, and 3043 remained after removing those represented in fewer than 10 taxa. These were concatenated into the final matrix whose aligned length is 2557548 base pairs, in which each taxon had on average ~2 million base pairs of sequence data (min. 940794, max. 2281787). Table 3 lists sequence data for four genes for 34 taxa, including bycatch sequence data for 25 UCE taxa and *Colopsus* spp. gathered from NCBI.

**Figure 1.**
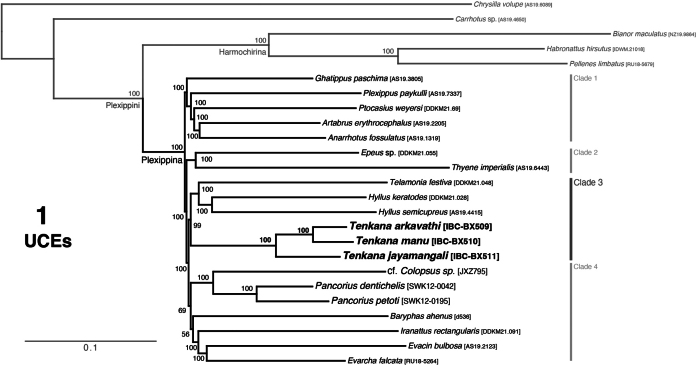
Maximum-likelihood tree from IQ-TREE analysis (best of 10 replicates) of a concatenated dataset of 3043 UCE loci. Numbers at the nodes are the percentage recovery of the clade based on 1000 bootstrap replicates. *Tenkana* is recovered as a sister genus to *Hyllus* and *Telamonia* in clade 3 and distantly from cf. *Colopsus* and *Pancorius* of clade 4.

**Figures 2, 3.**
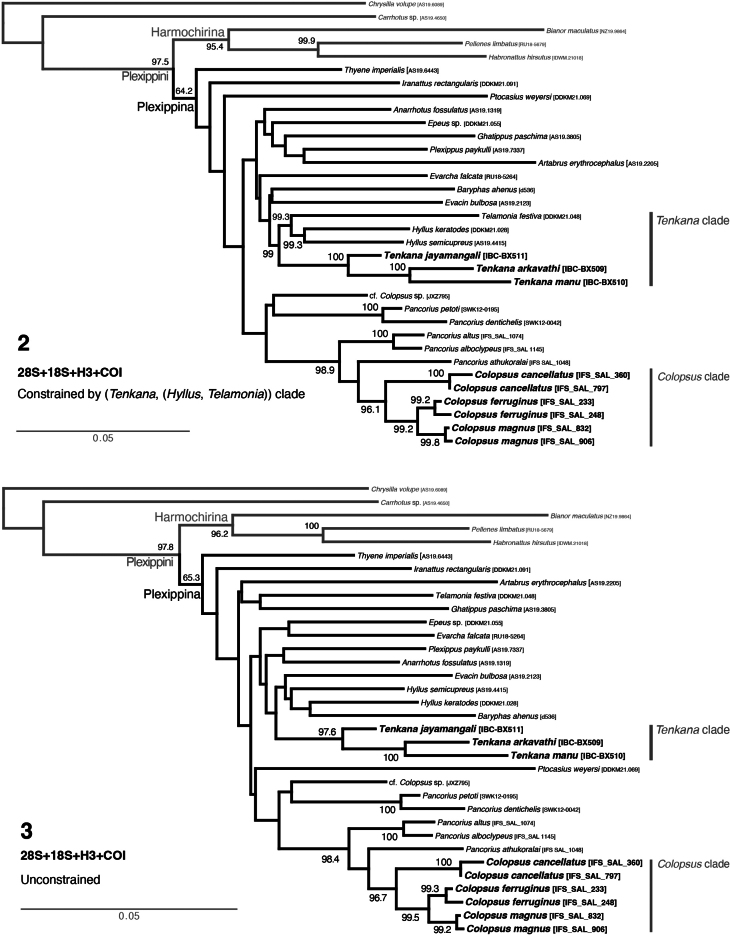
**2** Maximum-likelihood tree from IQ-TREE analysis (best of 10 replicates) constrained for Tenkana clade (using clade 3 of the UCE tree, see Fig. 1) **3** Maximum-likelihood tree from IQ-TREE analysis (best of 10 replicates) without the constraint. The trees are inferred from a gene partitioned, concatenated nuclear 28S, 18S, H3 and mitochondrial COI genes. Numbers at the nodes are the percentage recovery of the clade based on 1000 bootstrap replicates and nodes without numbers suggests that those clades were not recovered in bootstrap analysis. *Tenkana* is recovered distantly (see *Tenkana* clade) from the type species of *Colopsus*, *Colopsuscancellatus* and other Sri Lankan *Colopsus* (see *Colopsus* clade) in both constrained and unconstrained trees.

The phylogenetic results are shown in Figs 1–3. In the UCE phylogeny (Fig. 1), the broader phylogenetic structure and the structure within Plexippina are consistent with (Marathe et al. 2024a, 2024b) and show generally high bootstrap values at the nodes as expected for high volume data. The constrained four-gene phylogeny (Fig. 2) respects the constraint used for tree search. Thus, the relationship of *Tenkana* is recovered similarly to the UCE phylogeny as (*Tenkana*, (*Hyllus*, *Telamonia*)), showing high bootstrap values. However, the general relationships among plexippines in unconstrained and constrained four-gene phylogeny are less reliable, as reflected in the low bootstrap values.

*Tenkana* is nestled well within Plexippina as expected and recovered as the sister group to *Hyllus* C.L. Koch, 1846, and *Telamonia* Thorell, 1887 in clade 3 of the UCE phylogeny (see Fig. 1): (*Tenkana*, (*Hyllus*, *Telamonia*)). The type species of *Colopsus* and other members of the *cancellatus* group were found to be closely related to *Pancorius* Simon, 1902 (similarly to Kanesharatnam and Benjamin 2021). The *manu* species group is not closely related, however, to *Pancorius* and *Colopsus*. In the UCE phylogeny, it is recovered distantly from *Pancorius* and cf. *Colopsus* of clade 4 (see Fig. 1), while in unconstrained and constrained four-gene phylogenies, it is distant from *Pancorius*, cf. *Colopsus*, and the true *Colopsus* from Sri Lanka (Figs 2, 3). Their phylogenetic distance helps to explain the morphological differences between the *manu* group (non-glossy, non-elongate-bodied, tegulum with tegular lobe, medially located two ECPs) and the *cancellatus* group (glossy, elongate-bodied, without tegular lobe, laterally placed two ECPs).

Therefore, we propose *Tenkana* as a new genus to contain two species of the *manu* group currently placed within *Colopsus* and the new species described below.

### ﻿Taxonomic results

**Family Salticidae Blackwall, 1841**

**Subfamily Salticinae Blackwall, 1841**

**Tribe Plexippini Simon, 1901**

**Subtribe Plexippina Simon, 1901**

#### ﻿ Tenkana

Taxon classificationAnimaliaAraneaeSalticidae

Marathe, Maddison & Caleb gen. nov.

1F4CB40A-1F27-55B1-A21A-CCE377C1D504

https://zoobank.org/DE907A64-1CEC-4AE0-8976-929CCC31553D

##### Type species.

*Hyllusmanu* Caleb, Christudhas, Laltanpuii & Chitra, 2014.

##### Species included.

*Tenkanaarkavathi* (Caleb, 2022), comb. nov.; *Tenkanajayamangali* gen. et sp. nov.; *Tenkanamanu* (Caleb, Christudhas, Laltanpuii & Chitra, 2014), comb. nov.

##### Etymology.

‘Tenkana’ is a Kannada word meaning ‘south’. The name acknowledges that all known species of the genus are found in the southern part of the Indian subcontinent. The gender of the name is to be treated as feminine. The transliterations to different Indian languages are meant only for accessibility and do not represent required pronunciations or transliterations.

##### Diagnosis.

The phylogeny implies genetic diagnosability. Morphologically, *Tenkana* is a ground-dwelling plexippine with very robust first legs, recognisable by conspicuous pale bands under the ocular area ridge, often covering the entire surface and narrowing posteriorly on a rounded carapace. The teardrop-shaped abdomen has a broad median pale band. The stubby, non-glossy body of *Tenkana* distinguishes it from elongate, glossy *Colopsus* and from its closest relatives, glossy *Hyllus* and elongate *Telamonia*.

*Tenkana* may resemble *Hyllus* in sharing rounded body form, hair tufts behind ALEs, and membrane-accompanied embolus, but differs in epigyne (two ECPs in *Tenkana* vs none or reduced in *Hyllus*), and RTA (relatively delicate, narrow, short with pointed tip vs robust, broad with serrated broad tip). *Tenkana* can be confused with *Colopsus*, but they differ in embolus (membrane-accompanied in *Tenkana* vs membrane-lacking in *Colopsus*), tegular lobe (pronounced vs unpronounced or lacking), epigyne (medially located ECPs vs laterally located ECPs), and chelicerae (simple vs exaggerated).

##### Distribution.

The southern states of India (Andhra Pradesh, Karnataka, Kerala, Tamil Nadu, and Telangana) and the northern region of Sri Lanka.

##### Natural history.

*Tenkana* appears to be an exclusively ground-dwelling group. It is often found among relatively complex microhabitats of shaded short grasses with dry leaf litter in groves or relatively simpler microhabitats in open, sunny, sparse short grasses associated with rocky outcrops in relatively dry habitats. Its movements are reminiscent of those of the unrelated ground-dwelling *Stenaelurillus* jumping spiders (Aelurillini, Aelurillina).

#### ﻿ Tenkana jayamangali

Taxon classificationAnimaliaAraneaeSalticidae

Caleb & Marathe sp. nov.

2243FC58-BFD2-5100-8A8A-52B8F9ACA0F3

https://zoobank.org/C4C0B4D7-20BF-4566-959B-C015940AF4E6

Figs 4–7 , 8–15 , 16–24

##### Materials examined.

India • Karnataka, Tumakuru; 13.3843°N, 77.2069°E; 987 m a.s.l.; 23 April 2023; coll. Y.T. Lohit & B.G. Nisha. ***Holotype***: • ♂ (I/SP-48) in ZSIC, ***Paratypes***: • 1 ♀ (I/SP-49) in ZSIC; • 1 ♂ (IBC-BX512); • 1 ♀ (IBC-BX513) in NCBS. ***Paratype***: • 1 ♀ (IBC-BX511) in NCBS, 16 December 2023; coll. B.G. Nisha.

##### Etymology.

The specific epithet ‘jayamangali’, a noun in apposition, is the name of a river originating in Devarayanadurga, Tumakuru, where this species was observed for the first time.

##### Diagnosis.

The phylogenies recover *Tenkanajayamangali* as a sister species to *T.arkavathi* and *T.manu*. In the males of *T.jayamangali*, pale hairs occupy most of carapace surface area leaving small bald patch posteriorly, while in *T.arkavathi* and *T.manu*, pale hairs are gentler on carapace forming narrower bands on carapace laterally, tapering posteriorly. Ocular area of *T.jayamangali* covered with white hairs uniformly, while *T.arkavathi* has distinctive V-shaped bands and *T.manu* has bald ocular area. From ventrally, RTA can be seen extending much more laterally with slight bend sub-apically in *T.jayamangali*, while *T.arkavathi* with relatively short with prominent bend and *T.manu* with longer with no bend. Short sperm duct loop arising at 11 o’clock in *T.jayamangali*, while much longer sperm duct arising at 10 o’clock in *T.arkavathi* and *T.manu*). ECPs laterally placed *T.jayamangali*, while ECPs medially in *T.arkavathi* and *T.manu*).

##### Description.

**Figures 4–7.**
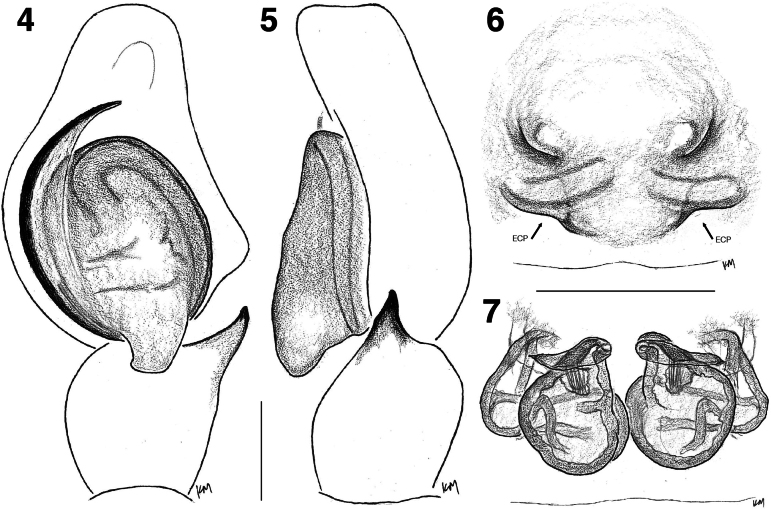
Genitalia of *Tenkanajayamangali* sp. nov. **4** male left palp, ventral view (holotype I/SP-48) **5** ditto, retrolateral view (holotype I/SP-48) **6** epigyne, ventral view (paratype IBC-BX511) **7** vulva, dorsal view (paratype IBC-BX511). Scale bars: 0.2 mm. **ECP**, epigynal coupling pocket.

**Figures 8–15.**
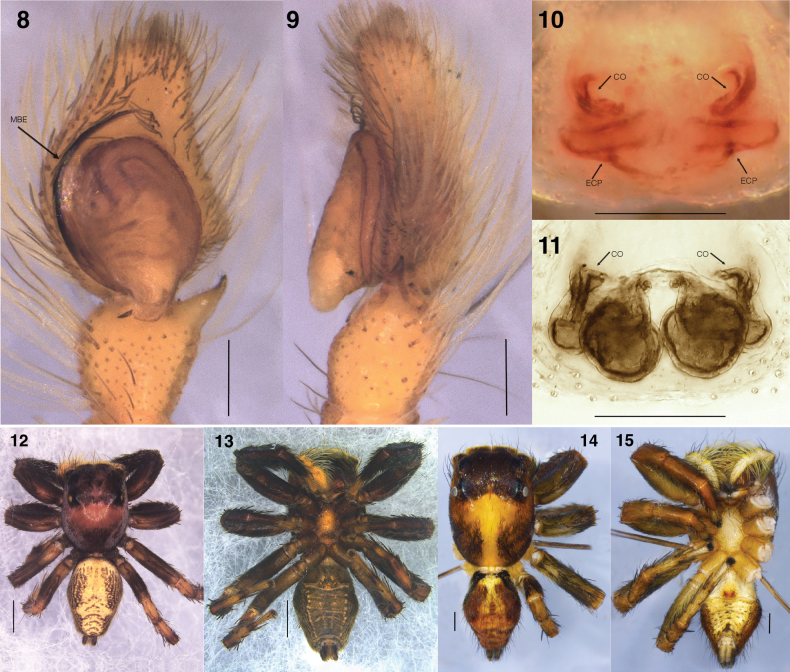
Photographs of genitalia and preserved bodies of *Tenkanajayamangali* sp. nov. Genitalia **8** male left palp, ventral view (holotype I/SP-48) **9** ditto, retrolateral view (holotype I/SP-48) **10** epigyne, ventral view (paratype IBC-BX511) **11** vulva, dorsal view (paratype IBC-BX511). Bodies: **12** male (holotype I/SP-48), dorsal view **13** ditto, ventral view **14** female (paratype IBC-BX511), dorsal view; 15, ditto, ventral view. Scale bars: 0.2 mm for genitalia; 1.0 mm for bodies. **CO**, copulatory opening; **ECP**, epigynal coupling pocket; **MBE**, membrane-bearing embolus.

♂ (I/SP-48 in ZSIC). Total length 5.18; carapace 2.66 long, 2.09 wide; abdomen 2.52 long, 1.77 wide. ***Carapace***, brown, white hairs laterally traversing posteriorly. In life, white hairs cover most of its surface area leaving small patch of bald black integument on thoracic slope. Tufts of thick bunch of hairs (‘eye lashes’) behind ALEs and below PMEs. In life, reddish-orange small hairs along the circumference of anterior eyes. ***Clypeus*** brown, length 0.19. ***Chelicerae*** brown. ***Legs*** brown, robust. Leg I and II ventrally fringed thickly with black hairs, but less densely on leg II. White leaf-like hairs interspersed prolaterally on patella and tibia of leg I; similar white hairs retrolaterally on femora of leg I and II and distal end and proximal and distal ends of leg III and IV femora. Leg measurements: I 5.66 (1.68, 1.16, 1.38, 0.88, 0.56); II 4.86 (1.62, 0.89, 1.05, 0.77, 0.53); III 5.54 (1.94, 0.94, 1.06, 0.95, 0.65); IV 5.56 (1.77, 0.76, 1.13, 1.19, 0.71). Leg formula 1432. ***Palp*** (Figs 4, 5, 8, 9) medium long membrane-accompanied embolus arising at 6 o’clock. Ovoid tegulum with prominent tegular lobe. RTA short, simple (Figs 5, 9). ***Abdomen*** brown and black mottlings on yellow integument, which is pronounced medially with band like appearance. Chevronous markings posteriorly. In life, bright orange coloured. Spinnerets brown, somewhat long.

♀ (IBC-BX511 in NCBS). Total length 5.24; carapace 2.53 long, 2.19 wide; abdomen 2.71 long, 1.83 wide. ***Carapace*** brown, more or less bald. White pale stripe posteriorly on thoracic slope. Tufts of thick bunch of hairs (‘eye lashes’) behind ALEs and below PMEs. In life, reddish-orange small hairs along the circumference of anterior eyes. ***Clypeus*** brown, length 0.16. ***Chelicerae*** brown. ***Legs*** brown, robust, largely bald. Leg measurements: I 5.36 (1.66, 1.05, 1.23, 0.85, 0.57); II 4.85 (1.60, 0.94, 0.97, 0.80, 0.54); III 5.68 (2.00, 1.02, 1.06, 0.98, 0.62); IV 5.57 (1.78, 0.82, 1.09, 1.21, 0.67). Leg formula 3412. ***Abdomen*** comparable as in male. In life, medial yellowish band surrounded by yellow and black mottlings. ***Epigyne*** (Figs 6, 7, 10, 11) medially located shallow two ECPs flanked by crescent shaped copulatory openings. Lamellar copulatory ducts join simple spermatheca ventrally.

##### Distribution.

In addition to the type locality, iNaturalist observations (e.g. Mohan 2024; Raj 2024) appearing to represent this species are recorded around Bengaluru, Karnataka.

##### Natural history.

*Tenkanajayamangali* was observed commonly in May; however, given the collecting of a female in December and iNaturalist observations, they may be adult year-round. *Tenkanajayamangali* were collected among dry leaf litter on the ground. A subadult was observed feeding on a bug nymph (Figs 23, 24).

**Figures 16–24.**
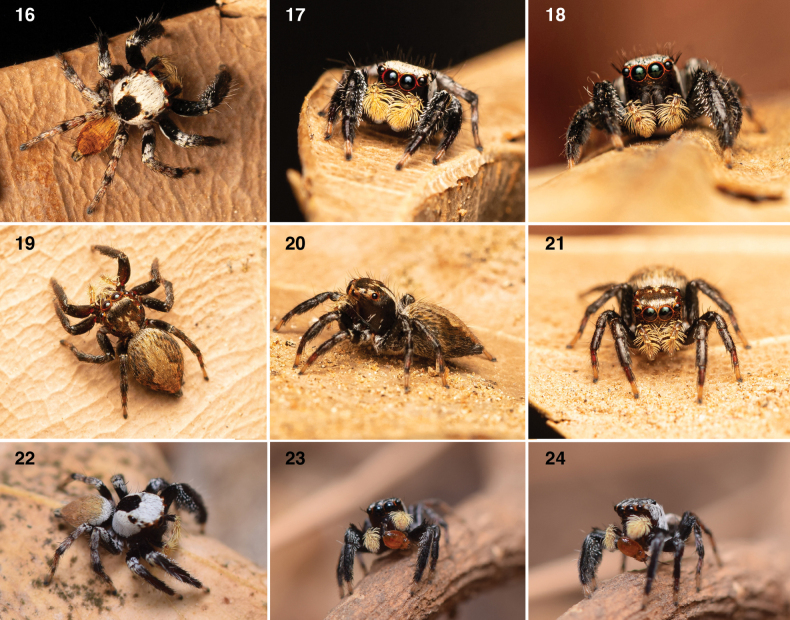
Habitus of *Tenkanajayamangali* sp. nov. **16–18** male **19–21** female **22** male **23–24** subadult male feeding on a bug nymph. Photo credit: Nisha B.G. (**16–21**) and Lohit Y.T. (**22–24**).

##### Discussion.

With *Tenkana*, the subtribe Plexippina now contains 36 genera; for India, the number of plexippines is 48 species in 19 genera (Maddison 2015; Marathe et al. 2024b; World Spider Catalog 2024). We continue to include *Colopsus* in the list of Indian plexippine genera, represented by *Colopsuspeppara* Sudhin, Sen & Caleb, 2023. *Colopsuspeppara* resembles *Tenkana* and *Pancorius* in body form; however, the features of the male palp could be attributed to *Colopsus* and *Pancorius* (simple round tegulum lacking tegular lobe and short embolus). What makes it puzzling is its epigyne, which has a medially located single ECP and does not match any of the three genera. The puzzling morphological features of *C.peppara*, along with a lack of clear synapomorphies for *Colopsus*, *Tenkana*, and *Pancorius*, compound the challenge of placing *C.peppara* definitively within a plexippine genus. Therefore, we propose maintaining the status quo until we can determine its placement using molecular data.

## Supplementary Material

XML Treatment for Tenkana

XML Treatment for Tenkana jayamangali

## ﻿Additional information

### Author ORCIDs

Kiran Marathe https://orcid.org/0000-0002-7364-3475

John T. D. Caleb https://orcid.org/0000-0002-9471-9467

Wayne P. Maddison https://orcid.org/0000-0003-4953-4575

B.G. Nisha https://orcid.org/0009-0006-7603-0785

Chinmay C. Maliye https://orcid.org/0009-0002-4050-9197

Y.T. Lohit https://orcid.org/0009-0007-8866-2226

Krushnamegh Kunte https://orcid.org/0000-0002-3860-6118

### Data availability

All of the data that support the findings of this study are available in the main text.
